# The Type 7 Serotonin Receptor, 5-HT_**7**_, Is Essential in the Mammary Gland for Regulation of Mammary Epithelial Structure and Function

**DOI:** 10.1155/2015/364746

**Published:** 2015-01-18

**Authors:** Vaibhav P. Pai, Laura L. Hernandez, Malinda A. Stull, Nelson D. Horseman

**Affiliations:** ^1^Department of Molecular and Cellular Physiology, University of Cincinnati, Cincinnati, OH 45267, USA; ^2^Center for Regenerative and Developmental Biology, Tufts University, Medford, MA 02155, USA; ^3^Department of Dairy Science, University of Wisconsin-Madison, Madison, WI 53706, USA; ^4^Department of Natural Sciences, Asbury University, Wilmore, KY 40390, USA

## Abstract

Autocrine-paracrine activity of serotonin (5-hydroxytryptamine, 5-HT) is a crucial homeostatic parameter in mammary gland development during lactation and involution. Published studies suggested that the 5-HT_7_ receptor type was important for mediating several effects of 5-HT in the mammary epithelium. Here, using 5-HT_7_ receptor-null (HT7KO) mice we attempt to understand the role of this receptor in mediating 5-HT actions within the mammary gland. We demonstrate for the first time that HT7KO dams are inefficient at sustaining their pups. Histologically, the HT7KO mammary epithelium shows a significant deviation from the normal secretory epithelium in morphological architecture, reduced secretory vesicles, and numerous multinucleated epithelial cells with atypically displaced nuclei, during lactation. Mammary epithelial cells in HT7KO dams also display an inability to transition from lactation to involution as normally seen by transition from a columnar to a squamous cell configuration, along with alveolar cell apoptosis and cell shedding. Our results show that 5-HT_7_ is required for multiple actions of 5-HT in the mammary glands including core functions that contribute to changes in cell shape and cell turnover, as well as specialized secretory functions. Understanding these actions may provide new interventions to improve lactation performance and treat diseases such as mastitis and breast cancer.

## 1. Background

The mammary gland cycles through waves of proliferation and regression associated with each pregnancy [1, 2]. This unique regenerative ability of the mammary gland is driven by the hormonal surges associated with pregnancy and lactation with each cycle consisting of a burst of alveolar epithelial growth and differentiation during pregnancy, followed by copious alveolar milk production during lactation, and concluding with massive epithelial apoptosis and remodeling during involution. The dynamic nature of the mammary gland also makes it vulnerable to breast diseases like mastitis and breast cancer. Such diseases arise due to the dysfunctioning of fundamental homeostatic regulatory mechanism that governs mammary gland development and function.

A number of recent studies implicate the regressing phase of the mammary gland (involution) in creating conditions facilitative for breast diseases including breast cancer [3–6]. During mammary gland involution, there is a systematic disassembly of the established lactation machinery, which is triggered by weaning-induced milk accumulation (milk stasis) within the glands and disruption of epithelial barrier function [7]. Local factors are largely implicated in inducing mammary involution [8–10]. One of the local factors synthesized and secreted by mammary epithelial cells is the monoamine serotonin (5-hydroxytryptamine, 5-HT) [11, 12]. 5-HT is potently induced upon milk stasis and is a crucial autocrine-paracrine regulator of involution. This action of 5-HT is conserved through many species [11–14].

Similar to other tissues, the bioactive level of 5-HT in the mammary gland is dynamically regulated by tryptophan hydroxylase (TPH), the 5-HT reuptake transporter (SERT), and monoamine oxidase (MaO) [11, 14–16]. 5-HT exerts its actions through 7 classes of receptors (5-HT_1–7_) encoded by 17 human genes [17]. The receptors are G-protein coupled with the exception of the ionotropic type 3 receptors (5-HT_3A–E_), which are ligand-gated cation channels. The mammary epithelium expresses multiple 5-HT receptors [15, 18, 19], with 5-HT_7_ having been shown to be important, based on* in vitro *studies [15, 18–20]. 5-HT_7_ is a G_s_-coupled receptor, which induces cAMP accumulation in the mammary epithelial cells upon 5-HT binding [15, 17, 18, 20]. 5-HT through 5-HT_7_ regulates mammary epithelial TJ* in vitro* [15, 20, 21]. However, the process of involution involves multiple events regulated simultaneously in addition to TJ regulation. Here, we use HT7KO mice to gain insights into the other cellular events regulated by 5-HT through 5-HT_7_.

Here, we show that HT7KO dams were inefficient at sustaining their pups, partly due to decrease in milk secretion necessary to sustain their pups. In addition, HT7KO epithelium failed to undergo morphological and physiological changes associated with the transition from lactation to involution. These findings are crucial in understanding the homeostatic mechanism employed by 5-HT in regulating epithelial morphological changes brought forth during transition from lactation to involution and may give possible insights into its role in creating conditions favorable for breast pathologies.

## 2. Results

### 2.1. HT7KO Dams Fail to Sustain Their Pups

*In vitro* experiments had been published indicating that 5-HT_7_ receptors are involved in mediating 5-HT actions in the mammary glands [15, 18, 20, 21]. To determine the* in vivo* physiological significance of 5-HT_7_ signaling for mammary gland development, we examined lactation in HT7KO mice. Wild type (WT) and HT7KO female littermates were mated to WT sires and their ability to bear litters and sustain pups was analyzed. There was no difference in the ability of HT7KO mice to get pregnant, gestate, and give birth to live pups. However, it was clear that the offspring of HT7KO mothers died at a high rate during the first few days of life. Because of the potential roles of 5-HT_7_ in the brain, maternal behavior was carefully observed. HT7KO mothers displayed normal maternal behaviors (nesting, crouching, and pup grooming), and the nursing behavior of the pups (nipple attachment and sucking) was also normal.

**Figure 1:**
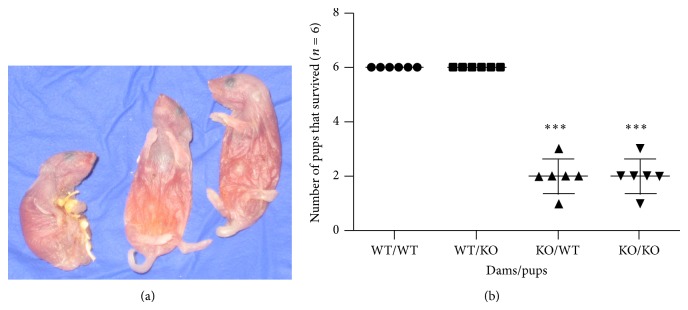
HT7KO dams are inefficient at sustaining their pups. (a) Image of dead WT pups from the HT7KO dam's cross-fostered litter showing the shriveled yet intact bodies of the pups suggesting their death due to insufficient nutrition. (b) This represents a graph of a number of pups (out of *n* = 6) that survived when fostered with WT or HT7KO dams. *N* = 6 dams was maintained for each group. The data was analyzed by two-way ANOVA. ^***^ *P* < 0.001.

To determine whether lactation was defective in the HT7KO mothers, WT offspring were cross-fostered onto either WT or HT7KO mothers on day one after birth. WT dams fostered WT pups with 100% survival during the first week of life. In contrast, 66% of the WT pups fostered with HT7KO dams died by day 4 after parturition (Figure 1). Each dam was kept with a small litter (*n* = 6 pups) so that the dams are not under much pressure for lactation. Even at such low lactation pressure, the pup survival was affected in the HT7KO suggesting inefficient lactation. Bodies of the dead pups were discovered intact indicating that pup deaths were caused by malnutrition and dehydration and not cannibalism (Figure 1(a)).

### 2.2. Epithelial Abnormalities in Lactating HT7KO Glands

Lactating mammary glands on day 10 postpartum were examined. In order to equalize lactation pressure on the dams, litter sizes were kept at 10 by replacing pups that died or were obviously weak. Histomorphologies of WT and HT7KO mammary glands were compared to explore possible reasons for the observed lactation defects and multiple abnormalities were observed.

**Figure 2:**
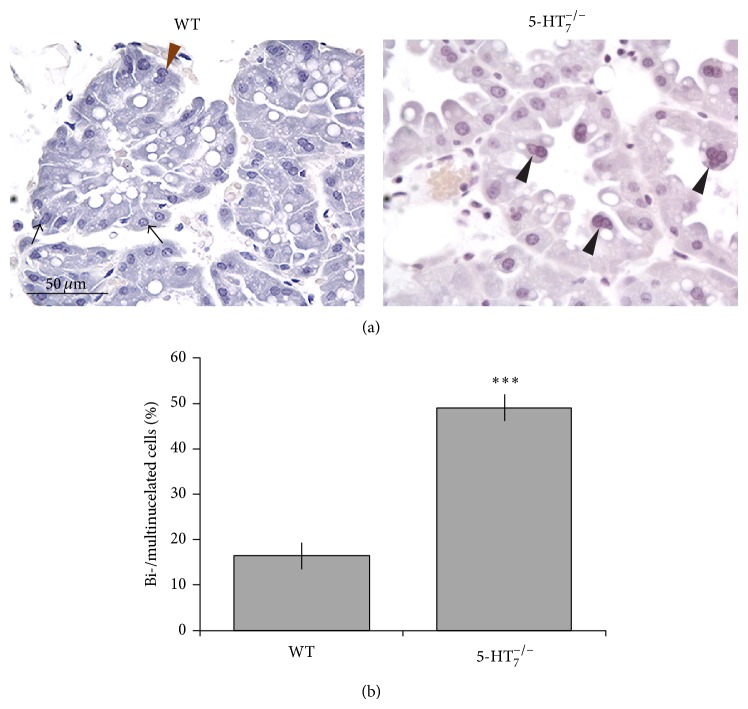
HT7KO lactating epithelium shows dysregulated cellular architecture. (a) WT and HT7KO glands were harvested during peak lactation (day 10) and stained with hematoxylin. Black arrows indicate mononucleated cells with basal nuclear localization. Brown arrowhead indicates binucleated cells with basal nuclear localization. Black arrowheads indicate the bi- and multinucleated cells that have nonbasal nuclear localizations. (b) Quantitative analysis of the multinucleated cells represented as % of alveolar cells that are bi-/multinucleated per alveolus. The sections of glands from various mice were counted. The data is represented as mean ± SEM. The data was analyzed using *t*-test. ^***^ *P* < 0.001.

The HT7KO epithelium contained numerous bi- and multinucleated cells (Figures 2(a) and 2(b), black arrowheads, *P* < 0.001), which were uncommon in the epithelium of WT type glands. Perhaps most strikingly, the basal positioning of nuclei, characteristic of normal columnar epithelia (Figure 2(a), black arrow), was disrupted in the majority of the HT7KO mammary epithelial cells. Nuclei were inconsistently located in the HT7KO cells, with many apical nuclei (Figure 2(a), black arrowheads), contributing to a scalloped appearance of the luminal epithelial surface. The unusual positioning of nuclei often, but not always, corresponded with multinucleated cells. About 50% of HT7KO alveolar cells were bi- or multinucleated (Figure 2(b)), and misplaced nuclei ranged from 28 to 60% in different alveoli. The luminal epithelial surface in lactating mammary glands is typically very dynamic, fimbriated, and populated by numerous lipid globules and secretory droplets. The luminal surfaces of 5-HT7KO glands appeared to be congested by comparison, consistent with the poor lactational performance of these dams. These observations suggested that 5-HT_7_ has important cellular functions that are essential for normal epithelial homeostasis within the lactating mammary glands.

### 2.3. Response of HT7KO Epithelium to Milk Stasis

**Figure 3:**
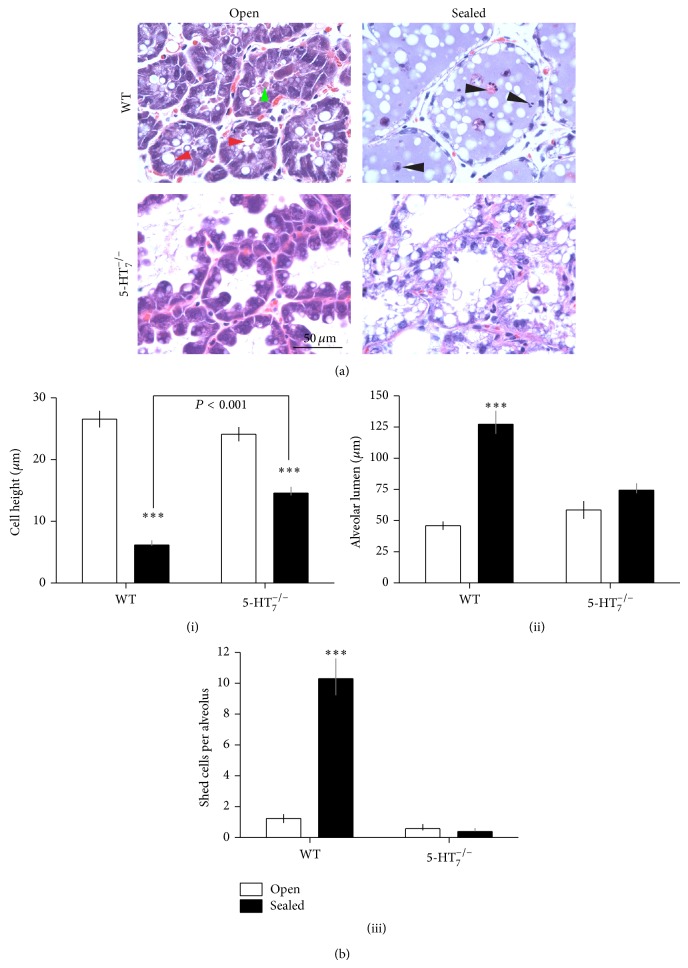
Lactating HT7KO mammary glands are morphologically altered and fail to undergo morphological transition associated with milk stasis induced involution. (a) A unilateral teat-sealing experiment was performed on WT and HT7KO lactating mice at peak lactation (day 10). Sealed and unsealed glands were harvested 48 hours (day 2) after sealing (*n* = 3). Representative contralateral (sealed and open) hematoxylin and eosin stained sections from number 4 glands are shown. Red arrowheads point to the secretory vesicles present within the mammary epithelial cells. Green arrowhead indicates secreted materials filling the alveolar lumen. Black arrowheads point to apoptotic shed cells. (b) Morphometric data on the mammary glands with respect to (i) cell height, (ii) alveolar lumen diameters, and (iii) number of shed cells per alveolus. The data was analyzed by two-way ANOVA. ^*^ *P* < 0.05, ^**^ *P* < 0.01, and ^***^ *P* < 0.001.

Glands of lactating dams were subjected to unilateral teat-sealing as a way of challenging epithelial homeostasis and establishing the participation of local regulatory mechanisms. Representative images from WT and HT7KO glands are depicted in Figure 3(a). Note that these sections were stained more deeply than those in Figure 2 so as to visualize structures in the sealed glands, which are less basophilic and therefore do not take stain as readily as actively lactating glands. WT lactating (WT-open) glands were characterized by tightly packed columnar epithelia lining the secretory alveolar units and the lumens were filled with secretory material (green arrowhead). The epithelial cells were highly basophilic and contained large and numerous secretory vesicles and lipid globules (red arrowheads), consistent with active biosynthetic and secretory activities. Three features that were obviously different from normal lactating epithelium were seen in the HT7KO lactating glands (Figure 3(a), HT7KO, open): (1) the free apical surface was scalloped, with bulbous or rounded tips, (2) the alveolar lumens were largely devoid of stained secretory material, and (3) the epithelial cells lining the alveoli had fewer and smaller vesicles and lipid globules. The other differences were in the nuclei distributions, as shown in Figure 2, which are difficult to see in Figure 3 because of the darker staining.

In response to milk stasis, the WT glands underwent the characteristic changes in morphology that have been documented during milk stasis [2, 22] (Figure 3(a)). The epithelium changed from being highly columnar to flattened (squamous transition) (Figures 3(a) and 3(b)(i)). Large amounts of secreted material (milk) accumulated within the alveolar lumens (Figure 3(a)) with a concomitant increase in the alveolar lumen diameters in comparison to lactating glands (Figures 3(a) and 3(b)(ii)). Typically, the lumens contained many shed cells undergoing cell death (black arrowheads, Figures 3(a) and 3(b)(iii)) [23]. In comparison, the sealed HT7KO glands failed to undergo the same set of morphological changes in response to milk stasis. The knockout epithelium failed to completely transition to a squamous configuration (Figures 3(a) and 3(b)(i), (ii)). The HT7KO epithelial cells had large vacuoles and/or lipid globules (Figure 3(a)), but the alveolar lumens appeared empty and the luminal spaces were not expanded (Figures 3(a) and 3(b)(ii)). The HT7KO glands did not show any increase in cells being shed into the lumens and undergoing cell death (Figures 3(a) and 3(b)(iii)).

## 3. Discussion

Intramammary 5-HT signaling is crucial for lactation homeostasis and transition into involution [2, 11, 12, 14–16, 24, 25]. Mammary epithelial cells express a variety of relevant genes that encode the machinery necessary to synthesize, secrete, and respond to 5-HT [11, 14, 15, 18–21]. Mammary epithelial cells express 5-HT_1D,2B,3A_ and _7_ receptors [18, 19].

Studies using pharmacological and* in vitro* approaches indicated that 5-HT_7_ regulated the epithelial tight junctions in mammary glands [14–16, 20]. To gain further insights into 5-HT_7_ functions at the tissue level, we used mice genetically deficient for 5-HT_7_ (HT7KO) [26] in the studies described here. HT7KO female mice conceived, progressed through pregnancy, and bore pups normally. However, the knockout dams had problems rearing their pups, as 66% of cross-fostered pups died from what appeared to be malnutrition and dehydration. The lactating HT7KO glands had significantly impaired milk production. In addition, during milk stasis, the HT7KO epithelium appeared to be highly vacuolated with empty alveolar lumens, further suggesting impaired secretion. TPH1 knockout (TPH1KO) mice showed a variety of defects in mammary epithelial homeostasis. When responding to prolactin, the TPH1KO glands were hypersecretory [11, 14]. Correspondingly, when hormone-stimulated glands were in milk stasis, the epithelium failed to transition to the typical squamous morphology. Many fewer cells were shed into the alveolar lumens, and therefore they did not undergo detachment-induced cell death (anoikis) [22, 23, 27]. These mammary phenotypes in TPH1KO mice were similar in many ways to the defects here in the HT7KO mice. An important difference between these models is that TPH1KO mice produced sufficient milk to nurse their litters, whereas HT7KO mothers did not. These differences suggest that unbalanced 5-HT_7_ signaling in the absence of type 7 receptors is detrimental to milk secretion.

A critical cell physiological event in mammary epithelial homeostasis is the opening of tight junctions in response to 5-HT-mediated p38 MAPK signaling [2, 15, 16, 20]. Because tight junctions are the only planar cell-cell junctions in the lactating mammary epithelium [28, 29], failure of tight junction disruption in HT7KO glands is sufficient to explain the defects observed during milk stasis.

### 3.1. 5-HT_7_ Mediates Morphological Transitions of Mammary Epithelium in Response to Milk Stasis

The lactating mammary gland is in a dynamic equilibrium. The production of milk must be balanced with the delivery of the milk to the nursing pups in order for the secretory properties of the gland to be maintained. In the absence of suckling, milk accumulation occurs and the gland is triggered to initiate events that ultimately lead to involution [2, 27]. Teat-sealing induces milk stasis, precipitating dramatic changes in the epithelial architecture in normal glands. In the epithelium transitions from a columnar to a squamous configuration, there is significant distention of the alveoli and many cells are shed and die. In contrast to normal glands, the HT7KO mammary epithelium failed to undergo the full range of morphological changes associated with milk stasis. The epithelium retained a columnar shape, and cell shedding was greatly reduced. Given that tight junctions are the only cell-cell junction in the lactating mammary epithelium, failure to disrupt tight junctions is sufficient to explain the lack of morphological changes and cell shedding. These findings correspond with the previous* in vitro *observations using selective 5-HT_7_ antagonist treatment. Inhibition of 5-HT_7_ resulted in enhanced epithelial membrane resistance and increased milk protein gene expression [15, 18, 19]. These results suggest that 5-HT acting through 5-HT_7_ is a critical mediator of the morphological and physiological transitions associated with cessation of milk synthesis.

### 3.2. 5-HT_7_ Mediates 5-HT Actions of Epithelial Cell Shedding and Cell Death

A characteristic feature of the involuting mammary gland is a marked increase in cell shedding and associated cell death [2, 27]. This has been proposed as a mechanism to rid the alveoli of spent cells and is also the most expedient way to eliminate cells that have been terminally differentiated as a means to prevent further milk accumulation. Previous studies using an* in vitro* model of functionally differentiated mammary epithelial cells [30] have shown that 5-HT induces epithelial cell shedding and cell death [21]. Interestingly, sealed HT7KO glands failed to exhibit any increase in epithelial shedding and cell death in comparison to lactating HT7KO glands. This demonstrates that 5-HT_7_ is critically involved in bringing about epithelial cell shedding and death.

A highly regulated cell turnover is critical in maintaining the secretory properties of the terminally differentiated lactating mammary epithelium. At peak lactation (day 10) in normal mammary glands, a small number of binucleated cells were observed. In contrast, the HT7KO lactating glands had dramatically increased numbers of binucleated (and multinucleated) cells (50%). In addition to the increased number of cells with multiple nuclei, it is important to note the disruption of the basal placement of the nuclei within the cells. These observations demonstrate that loss of 5-HT_7_ results in the dysregulation of cell renewal, not only in the number of cells undergoing nuclear division but also in fundamental differences in the intracellular organization within the cells.

A similar phenotype of multinucleated, highly vacuolated mammary epithelial cells is observed with overexpression of the E3 ubiquitin ligase protein MDM2 [31]. MDM2 overexpression in mammary epithelial cells resulted in the uncoupling of S-phase from mitosis, causing multiple rounds of DNA synthesis without cell division. Also, MDM2 is closely associated with suppression of cell death through inactivation of p53 tumor suppressor [31–33]. Interestingly, MDM2 expression is suppressed by increased intracellular cAMP levels [32]. 5-HT_7_ activation results in increased intracellular cAMP in the mammary epithelial cells [15, 20]. This in turn may suppress MDM2. Conversely, the absence of 5-HT_7_ activation, as is the case in 5-HT_7_^−/−^ mice, may result in sustained increases of MDM2 and increased cell survival. This dysregulation of MDM2 expression may then be a first step towards transformation and neoplastic growth within the mammary epithelium. This is consistent with other observations where MDM2 overexpression is linked to breast tumors and hyperplastic alveolar nodules in mice and also human breast cancers [31, 34]. The activation of 5-HT_7_, therefore, may serve as a deterrent against transformation of mammary epithelial cells. This hypothesis is supported by our previous observations on the complex role of 5-HT in breast cancers [18]. Our* in vitro *observation of 5-HT_7_ receptor-mediated suppression of cell growth/division lends further support to this hypothesis [18]. In addition, absence of 5-HT_7_ could result in enhanced activation of the 5-HT_2B_ receptor, which has been shown to have proliferative effects in other organ systems [35, 36]. Hence, it is conceivable that a lack of 5-HT signal through 5-HT_7_ (growth inhibitory, cell shedding, and cell death) leads to a shift in the balance of epithelial turnover, resulting in a multinucleated, overproliferating, and nonshedding epithelium.

## 4. Conclusions

In conclusion, the studies reported here provide valuable insights into the functions of 5-HT_7_ receptor signal in mediating various actions of 5-HT. Through the use of HT7KO mice, we demonstrate that the contribution of this receptor is crucial in maintaining the lactation capacity of the mammary gland. The 5-HT_7_ receptor is involved in regulating epithelial cell growth, shedding, and cell death. The regulation of epithelial tight junctions is likely to be the mechanistic common denominator in these phenotypes. Understanding how 5-HT_7_ downstream signals are able to regulate many crucial functions will be critical in making inroads towards achieving better regulation of mammary gland development, lactation, nursing, and understanding its involvement in breast pathologies including breast cancer.

## 5. Materials and Methods

### 5.1. Animal Studies

HT7KO mice (C57BL/6J background) were obtained from The Scripps Research Institute (Hedlund et al.) [26]. Mice were maintained on a L, D: 14, 10 daily light, darkness cycle with* ad libitum* food and water. Female HT7KO mice obtained by heterozygous crosses were used during lactation. Wild type (defined as homozygous +/+ and heterozygous +/−) littermates were phenotypically normal and were used as controls. To perform teat-sealing experiments, mice upon reaching peak lactation (day 10) were lightly anesthetized by isoflurane inhalation. The nipples on the left side of the body were exteriorized and sealed using a suture and surgical glue. The glands on the right side of the body remained open to serve as contralateral lactation controls [11, 22]. The dams were returned to their pups and visual confirmation of pups resuming suckling was obtained. Pups were allowed to nurse for 48 hrs after teat-sealing, at which time the dams were sacrificed using CO_2_ inhalation followed by cervical dislocation and the glands harvested and stored in 4% paraformaldehyde at 4°C overnight, then placed in 70% ethanol, and processed for tissue sectioning and hematoxylin and eosin staining.

### 5.2. Immunostaining

Mammary glands were harvested and fixed in 4% paraformaldehyde, followed by paraffin embedding. The samples were sectioned at 4 *μ*m and used for staining. The sections were permeabilized in 0.1% Triton-X-100. The following stains were used: hematoxylin and eosin. Images were collected using an Olympus Microscope.

### 5.3. Data Analysis

Data analysis was conducted using Prism, version 5.0b (GraphPad Software, San Diego, CA). An *n* = 6 was maintained for mice in each group, unless otherwise mentioned. All graphed results are represented as mean ± SEM. One-way ANOVA with Tukey's post hoc test or two-way ANOVA with Bonferroni's post hoc test was used to analyze the data. A probability level of 0.05 was considered as significant. ^*^ *P* < 0.05, ^**^ *P* < 0.01, and ^***^ *P* < 0.001.

### 5.4. Ethics Statement

All experiments were conducted under protocols that were approved (#05-01-11-01) by the Institutional Animal Care and Use Committee at University of Cincinnati.

## Abbreviations

5-HT: 5-hydroxytryptamine, serotonin
5-HT_7_: Type 7 serotonin receptor protein
SERT: Serotonin reuptake transporter
TPH: Tryptophan hydroxylase
TJ: Tight junctions.
